# Diurnal Rhythmicity of Autophagy Is Impaired in the Diabetic Retina

**DOI:** 10.3390/cells9040905

**Published:** 2020-04-07

**Authors:** Xiaoping Qi, Sayak K. Mitter, Yuanqing Yan, Julia V. Busik, Maria B. Grant, Michael E. Boulton

**Affiliations:** 1 Department of Ophthalmology and Visual Sciences, University of Alabama at Birmingham, AL 35294, USA; xqi@uabmc.edu (X.Q.); sayakmitter@gmail.com (S.K.M.); mariagrant@uabmc.edu (M.B.G.); 2 Department of Neurosurgery, The University of Texas Health Science Center, Houston, TX 77030, USA; Yuanqing.Yan@uth.tmc.edu; 3 Department of Physiology, Michigan State University, East Lansing, MI 48824, USA; busik@msu.edu

**Keywords:** diurnal rhythm, autophagy, retina, diabetes, diabetic retinopathy

## Abstract

Retinal homeostasis is under both diurnal and circadian regulation. We sought to investigate the diurnal expression of autophagy proteins in normal rodent retina and to determine if this is impaired in diabetic retinopathy. C57BL/6J mice and Bio-Breeding Zucker (BBZ) rats were maintained under a 12h/12h light/dark cycle and eyes, enucleated over a 24 h period. Eyes were also collected from diabetic mice with two or nine-months duration of type 1 diabetes (T1D) and Bio-Breeding Zucker diabetic rat (BBZDR/wor rats with 4-months duration of type 2 diabetes (T2D). Immunohistochemistry was performed for the autophagy proteins Atg7, Atg9, LC3 and Beclin1. These autophagy proteins (Atgs) were abundantly expressed in neural retina and endothelial cells in both mice and rats. A differential staining pattern was observed across the retinas which demonstrated a distinctive diurnal rhythmicity. All Atgs showed localization to retinal blood vessels with Atg7 being the most highly expressed. Analysis of the immunostaining demonstrated distinctive diurnal rhythmicity, of which Atg9 and LC3 shared a biphasic expression cycle with the highest level at 8:15 am and 8:15 pm. In contrast, Beclin1 revealed a 24-h cycle with the highest level observed at midnight. Atg7 was also on a 24-h cycle with peak expression at 8:15 am, coinciding with the first peak expression of Atg9 and LC3. In diabetic animals, there was a dramatic reduction in all four Atgs and the distinctive diurnal rhythmicity of these autophagy proteins was significantly impaired and phase shifted in both T1D and T2D animals. Restoration of diurnal rhythmicity and facilitation of autophagy protein expression may provide new treatment strategies for diabetic retinopathy.

## 1. Introduction

Macroautophagy (hereafter referred to as autophagy) is an evolutionarily conserved cellular catabolic mechanism that facilitates the degradation of damaged cellular organelles and proteins, by targeting them to the lysosomes and recycling the macromolecules for the rebuilding of cellular machinery [1,2,3]. Autophagy undergoes rhythmic variation in accordance with circadian patterns of rest/activity and feeding in adult mammals [4]. Dysregulated autophagy has been implicated in several neurodegenerative disorders, hepatitis, cancer, aging associated diseases and in the general aging process [5,6,7,8,9]. Recently, a growing body of evidence indicates that dysregulated autophagy is also linked to diabetes [10,11,12,13].

The disruption of circadian rhythm has a profound negative impact on health and is associated with elevated risk for several diseases [14]. The physiological relevance of an altered circadian rhythm in diabetes is evidenced by the observation of a high incidence of myocardial dysfunction, acute coronary syndrome, sudden cardiac death, and ischemic stroke in diabetics during the night, compared to a higher frequency during the day in non-diabetics [15,16,17,18]. In diabetic conditions, Bmal1 and Clock are inactivated, causing deregulated glucose homeostasis and suppressed diurnal variation in glucose and triglycerides, along with reduced gluconeogenesis [19]. Streptozotocin (STZ)-induced type 1 diabetes (T1D) in mice exhibits altered phase of the circadian clock in the heart [20] and a significant reduction of circadian sensitivity to low-intensity light in the retina [21]. Furthermore, STZ-mice develop a deficiency in their ability to re-entrain the circadian rhythm when subjected to a phase advance of the 12L/12D cycle [21]. Bio-Breeding Zucker diabetic rat (BBZDR/Wor) and Goto-Kakizaki rat type 2 diabetes (T2D) models also show impairment of the molecular clock, suggesting that the disruption of the circadian clock is a common phenomenon in both T1D and T2D [15,22].

Several hallmarks of diabetic retinopathy can be recapitulated in rodent retina deficient of clock genes [15,23]. Reduced tube formation and increased senescence of endothelial cells coupled with impaired progenitor-mediated repair is observed in Per2 mutant mice, emphasizing the importance of the circadian clock in retinal homeostasis [23]. Recent studies on autophagy in the retina have shed light on the association of key molecules of the autophagic pathway with phagocytosis of photoreceptor outer segments (POS) by the retinal pigmented epithelium (RPE) [24,25]. Photoreceptor disk shedding has been widely reported to exhibit diurnal rhythmicity in the retina [26,27] and the tight coupling of phagocytic ingestion and autophagic degradation of the POS to this diurnal rhythm is a critical aspect of retinal homeostasis [28,29].

The role of the peripheral clock and diurnal variation on the regulation of autophagy in the normal and diabetic rodent retinas and the fate of autophagy in the diabetic retina remain unexplored. Understanding these control mechanisms may help find effective treatments for diabetic retinopathy. In this study, we demonstrate that the spatial distribution and temporal expression of autophagy proteins show a diurnal rhythm and that this is depressed and phase shifted in the diabetic retina.

## 2. Materials and Methods

### 2.1. Experimental Animals

All animal procedures were performed in accordance with a) protocols approved by the Institutional Animal Care and Use Committees at University of Florida, Gainesville, FL, USA (#CR-201106001), Michigan State University, Lansing, MI, USA (#Busik09/14-160-00, and Indiana University, Indianapolis, IN, USA (#10574 MD/R), b, the National Institutes of Health Guide for Care and Use of Laboratory Animals and c) the ARVO Statement for the Use of Animals in Ophthalmic and Vision Research. Male C57BL/6J mice were purchased from Jackson Labs at 6 weeks of age and male BBZDR/wor T2D rats and lean heterozygote nondiabetic control littermates were obtained from biomedical research models (BRM, Inc.) Worcester, Massachusetts at 5 months of age. C57BL/6J mice (n = 240) and BBZ rats (n = 75) were maintained on a standard 12/12 h light/dark cycle (6.00 am lights on/6.00 pm lights off). T1D was induced in eight-week-old C57BL/6 mice (n = 120) by five consecutive intraperitoneal injection of freshly prepared streptozotocin (STZ) solution with the concentration of 40mg/kg body weight in 0.1M/citrate buffer, pH 4.5 as previously described [30,31]. The control group received sodium citrate buffer (vehicle) alone. Diabetes was confirmed by two consecutive blood glucose levels >240 mg/dl, using the AlphaTrak^®^ blood glucose monitor and test strip system (Abbot laboratories, Irvine, CA, USA), according to the manufacturer’s instruction. No animal used in this study required insulin injections. Eyes from one group were collected at 2 months following establishment of diabetes and the second cohort 9 months after diabetes induction (the age of the animals at these time points being 4 and 11 months, respectively). We also investigated inbred Bio-Breeding Zucker (BBZDR)/wor T2D rats (n = 37) and age-matched controls (n = 38) [15]. BBZDR/wor rats spontaneously became diabetic at ~10 weeks of age and were maintained for 4 months after the onset of diabetes. A second group of C57BL/6J mice (n = 24) were dark adapted for 48 h before sacrifice and referred to as being on the dark/dark cycle. In all groups, animals were euthanized by deep anesthesia with isoflurane followed by cervical dislocation and eyes were immediately enucleated every 2 or 3 h over a 24 h period and prepared for immunofluorescence staining. For animals in the dark cycle, euthanasia and enucleation were performed in a dark room under a red safe light. For each set of experiments, all animals were started at the same time point to clearly identify any phase shift in peak or trough of ATG expression. The number of biological replicates of the enucleated eyes at each time point is n = 10 for mice and n = 3 for rats. Only one eye from each animal was used in the assessment.

### 2.2. Immunofluorescence Microscopy

Eyes were processed for standard paraffin embedding and 4 µm sections were prepared. Rodent Decloaker (Biocare Medical LLC, Concord, CA, USA Catalog# RD913L) was used to unmask antigens and non-specific binding was blocked with 10% normal goat sera and 5% BSA for 1 h at room temperature. Sections received either mouse monoclonal anti-Beclin1 (BD Transduction Lab, San Jose, CA, USA, Cat#612112), or rabbit polyclonal antibodies against Atg7, Atg9 and LC3 (provided by Dr. Dunn, Department of Anatomy and Cell Biology, University of Florida, Gainesville, FL, USA), diluted in phosphate buffered saline (PBS) with 1% normal goat sera plus 1% bovine serum albumin (BSA) (Beclin1 1:20; Atg7- 1:300; Atg9 and LC3 - 1:100). After washing, sections were incubated with an appropriate FITC-conjugated secondary antibody for 1 h. In some sections, colocalization of autophagy proteins within the retinal vasculature was confirmed by dual staining with the endothelial cell marker, TRITC-agglutinin (Vector Labs, Burlingame, CA, USA, Cat#RCA120). Sections were covered with Vectashield mounting medium/DAPI (Vector Labs, Inc.). Sections were viewed using a Zeiss Axioplan 2 Upright Fluorescence Microscope with Qimage/QCapture software Version 8 (QImaging Corporate, Surrey, British Columbia, Canada). Omission of the primary antibody was the baseline control and all the fluorescence photographs were obtained under the same scaled conditions.

### 2.3. Grading of Immunostaining

Three independent masked observers, using a previously described grading system [32,33], graded the intensity of the immunoreactivity for each antibody in transverse retinal sections. The intensity of labeling was graded qualitatively as: 10, strong bright intense immunoreactivity, 9, bright intense; 8, uniformly intense; 7, patchy and intense; 6, uniform and moderate; 5, patchy and moderate; 4, uniform and weak; 3, patchy and weak; 2, uniform and very weak; 1, patchy and very weak; and 0, none. A mean score ±SEM for each group was determined from the scores of all graders for each retina structure.

### 2.4. Trypsin Digestion and the Detection of Superoxide

The retinal vasculature was prepared as previously described [34]. Briefly, mouse eyes were fixed overnight in 4% paraformaldehyde, freshly made in PBS. The retinas were dissected from the eye cups, kept in water overnight, and digested in 3% trypsin (Invitrogen-Gibco, Grand Island, NY, USA) for 3 h at 37 °Celcius. The tissue was mounted carefully on a glass slide under a dissection microscope. The tissue was stained with PAS-H&E (periodic acid Schiff–hematoxylin and eosin; Gill No.3; Sigma-Aldrich), according to the instruction manual. The images were taken using a Zeiss light microscope equipped with a digital camera (AxioCam MRC5, Axiovert 200; Carl Zeiss Meditec, Inc., Dublin, CA, USA), using 20X objective lenses. Eight to ten representative fields from each quadrant of the retina were imaged and the number of acellular capillaries per square millimeter of retina were quantified.

For detecting the reactive oxygen species (ROS), the superoxide indicator, hydroethidine, was used to detect the production of superoxide radicals, as previously described [35]. Superoxide oxidizes hydroethidine to yield a red fluorescent signal at approximately 600 nm. Mice received two intraperitoneal injections, 15 min apart, of freshly prepared hydroethidine (20 mg/kg; Invitrogen) and were euthanized 18 h after injection. The fluorescence intensity was measured in the neural retina using a fluorescent plate reader (BioTek, Winooski, VT, USA) and a spectrofluorometer (FLUOstar Optima; BMG Labtechnologies, Cary, NC, USA). The relative fluorescence intensity was calculated by normalizing to protein concentration.

### 2.5. Statistical Analysis

The diurnal data are time series data, and single cosine analysis was employed to fit the data. The data was considered as diurnal oscillation by a zero-amplitude test, with a *p*-value less than 0.05. All experiments were assessed by comparing two groups mean scores from control animals and diabetic animals using a Student’s *t*-test, plus ANOVA for multiple comparisons by using Prism^®^ statistical software ver. 5 (GraphPad, La Jolla, CA, USA) with differences of *p* value < 0.05 were considered statistically significant. Results are expressed as mean ± SEM.

## 3. Results

### 3.1. Autophagy Proteins Exhibit Diurnal Expression/Localization in Normal Mouse Neural Retina

**Figure 1:**
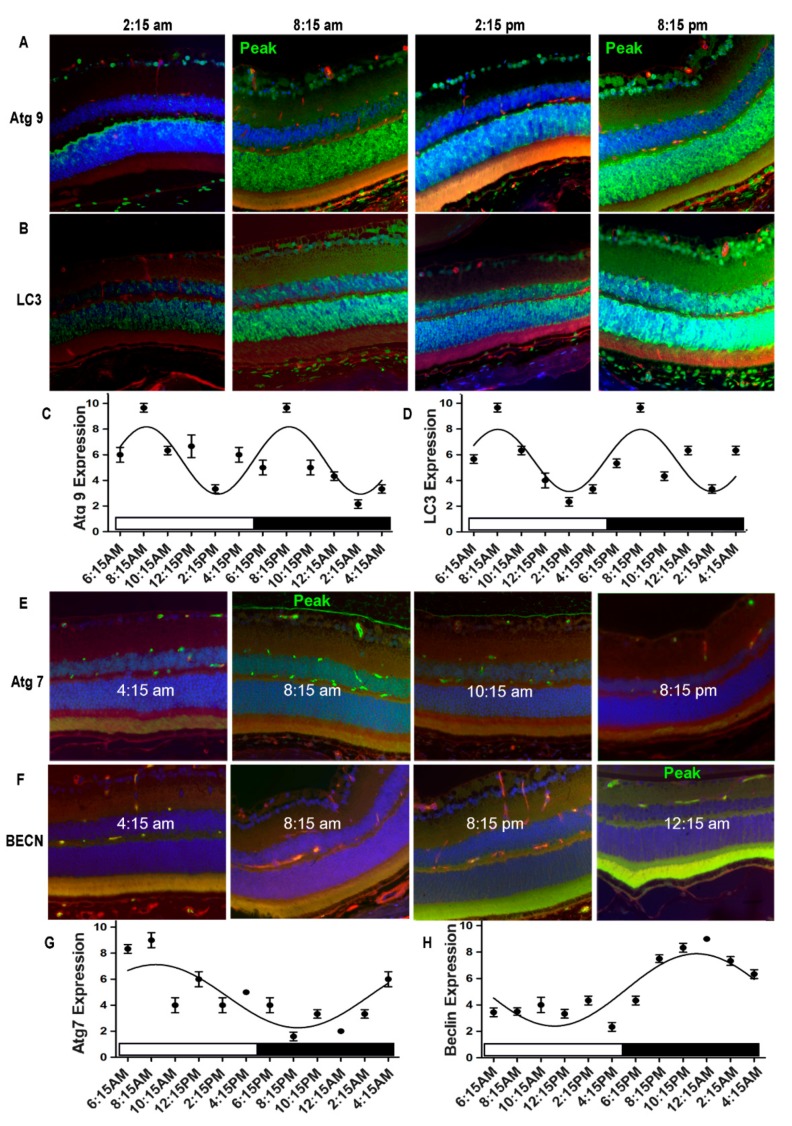
Autophagy proteins exhibited diurnal expression/localization in normal mouse neural retina. Retina were harvested every 2 h over a 24 h time period. Antibodies for autophagy proteins (ATGs) ATG9, Microtubule-associated protein 1A/1B-light chain 3 (LC3), ATG7 and Beclin1 (BECN) were used to detect respective protein expression (green) in the retina, Tetramethylrhodamine (TRITC) agglutinin for vessels (red) and DAPI (blue) shows nuclear staining. Distinct diurnal rhythmic patterns of the autophagic proteins Atg9 and LC3 expressions (**A**,**B**) revealed a biphasic diurnal cycle (12/12 h), with peaks at 8:15 AM and 8:15 PM and lowest levels at 2:15 AM and 2:15 PM (**C**,**D**). Atg7 and Beclin1 (BECN) expressions (**E**,**F**) were on a monophasic 24 h cycle. Atg7 expression peaked at 8:15 AM and lowest levels were at 10:15 AM. The peak of Beclin1 expression was at midnight and lowest levels were observed during the late morning (**H**). All animals were maintained in a standard 12/12 h light/dark phase with lights ON at 6:00 AM and lights OFF at 6:00 PM. The error bars in the diurnal plots represent the mean+SEM and diurnal oscillation had a *p*-value less than 0.05.

**Figure 2:**
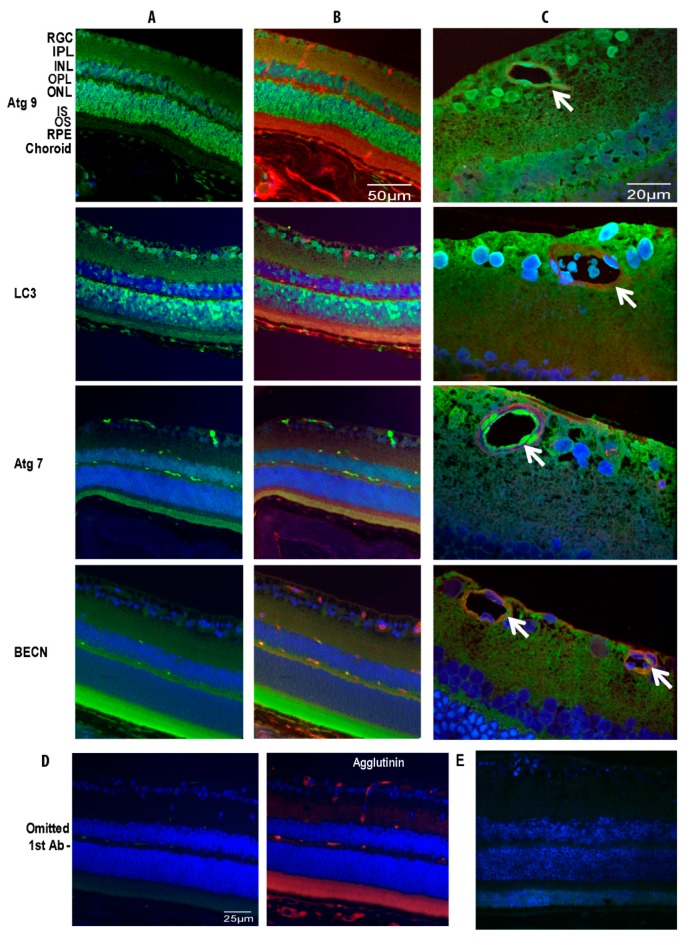
Localization of autophagy proteins in normal mouse retina and vasculature. Animals were kept in tight 12/12-h light/dark cycle before the experiment. Antibodies for ATG9, LC3, ATG7 and Beclin1 (BECN) were used to detect respective protein expression (green) in the retinas with different staining patterns and DAPI was used to stain nuclei (blue) (**A**). Agglutinin, an endothelial cell marker, conjugated with TRITC (red) was used to co-localize with antibodies specific to individual autophagy proteins (FITC) in the retinal vasculature (**B**). High magnification images demonstrated autophagy protein localization to the endothelial cells and pericytes of the retinal vasculature (**C**). No fluorescence was observed with omission of the primary antibody in the mouse retina. Using agglutinin staining, the section displayed retinal vascular patterns with normal architecture (**D**). Omitted autophagy primary antibody in rat retina also was negative (**E**). The arrows indicate autophagy protein localization to retinal vessels. RGC, retina ganglion cell; IPL, inner plexiform layer; INL, inner nuclear layer; OPL, outer plexiform layer; ONL, outer nuclear layer; IS, inner segment; OS, outer segment; RPE, retinal pigment epithelium.

To determine if autophagic protein expression in the neural retina had an intrinsic diurnal rhythm, we assessed the expression and localization of four autophagy proteins, Atg7, LC3, Atg9 and Beclin1 in the normal retinas of young mice (age = 3 months) that were maintained under standard 12 h light/12 h dark conditions. When we examined the tissue at two-hour intervals over a 24 h period, these proteins exhibited not only a distinct diurnal rhythm, but remarkably showed distinct staining patterns across the layers of the neural retina in mice (Figure 1 and Figure 2). Atg9 and LC3 exhibited a biphasic 12/12 h circadian cycle with zenith at 8:15 AM and 8:15 PM and nadir at 2:15 AM and 2:15 PM (*p* < 0.05) (Figure 1A–D). By contrast, Atg7 and Beclin1 expression showed a monophasic rhythm and the overall expression of these proteins was much lower than either Atg9 or LC3 (Figure 1E–H). The expression of Atg7 started to rise at 4:15 AM, peaked at 8:15 AM and gradually decreased until 10:15 AM (*p* < 0.05) (Figure 1E–G). Beclin1 expression was highest at around midnight and reached lowest levels at midday (*p* < 0.05) (Figure 1F–H). Omission of the primary antibody showed no staining in mouse retina (Figure 2D).

An assessment of the staining pattern of the proteins at zenith revealed that Atg9 and LC3 were localized throughout the retina, predominantly within the retinal ganglion cell (RGC) layer, inner (INL) and outer nuclear layer (ONL) (Figure 2A,B). Atg7 was only weakly localized to the neural retina in both the inner and outer nuclear layers and showed stronger localization in the photoreceptor outer segment. Beclin1 staining was detected throughout the retina, with highest intensity in the inner and outer plexiform layers and in the photoreceptors. Closer observation of the retina revealed that ATG7 and 9 showed remarkably strong association within the inner retinal vasculature (Figure 2C).

Using dual staining for endothelial cells and autophagy proteins, we confirmed that Atg9, LC3, Atg7 and Beclin1 were all expressed in both the inner and outer retinal plexus of the retinal vasculature (Figure 2C).

### 3.2. Autophagic Activity in the Retina Is Tightly Entrained by Light

**Figure 3:**
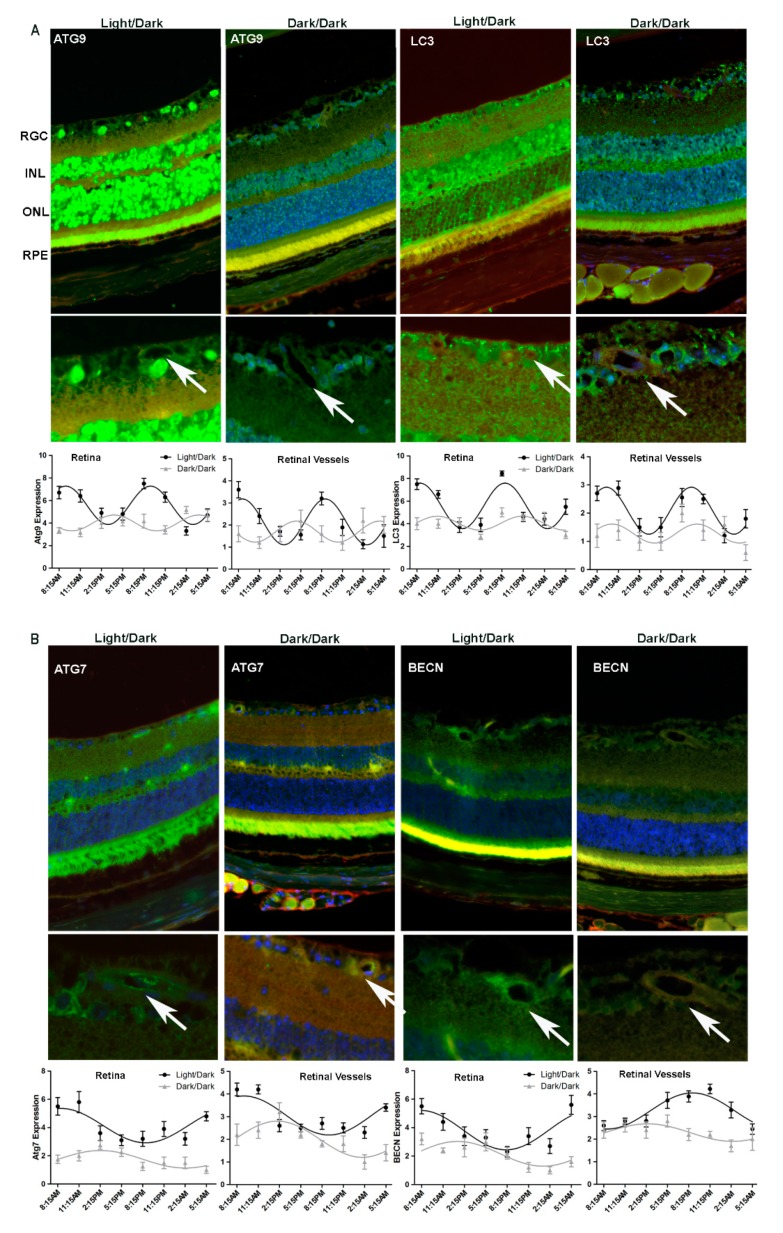
Immunohistochemistry confirmed alterations in diurnal patterns of autophagic gene expression upon light cycle disruption. Mice were separated into two groups. One group was kept under 12/12-h light/dark cycle and the other group was kept in the dark for 48-h (dark/dark), before being euthanized. Samples were collected every 3 h for 24 h and were analyzed by immunohistochemistry for autophagic markers (**A**) ATG9 (**B**) LC3 (**C**) ATG7 and (**D**) Beclin1 (BECN). Autophagy protein expression is green, TRITC agglutinin for vessels red and DAPI nuclear staining blue. The arrows indicate autophagy protein localization to retinal vessels. RGC, retina ganglion cell; INL, inner nuclear layer; OPL, outer plexiform layer; ONL, outer nuclear layer; RPE, retinal pigment epithelium. The error bars in the circadian plots represent the mean + SEM and diurnal oscillation had a *p*-value less than 0.05.

We repeated immunohistochemistry of ATG9, ATG7, LC3 and Beclin1 in the retina (and the retinal vasculature) of 48 h dark adapted mice and compared the results to those of 12/12 h light/dark adapted mice. The immunohistochemistry data indicated that the periodic oscillations, as well as overall levels of the autophagic proteins ATG9 and LC3, were attenuated in the 48 h dark adapted mice retina when compared to the 12/12 h dark/dark controls, both in the retina and the vasculature (Figure 3A). Similarly, peaks in Atg7 and Beclin1 were phase shifted compared to the light/dark controls and overall expression levels were reduced (Figure 3B). We concluded that diurnal oscillations in autophagic activity in the murine retina depend, at least in part, on light-effected entrainment.

### 3.3. Diurnal Rhythmicity of Expression of Autophagic Proteins Is Dampened in T1D

**Figure 4:**
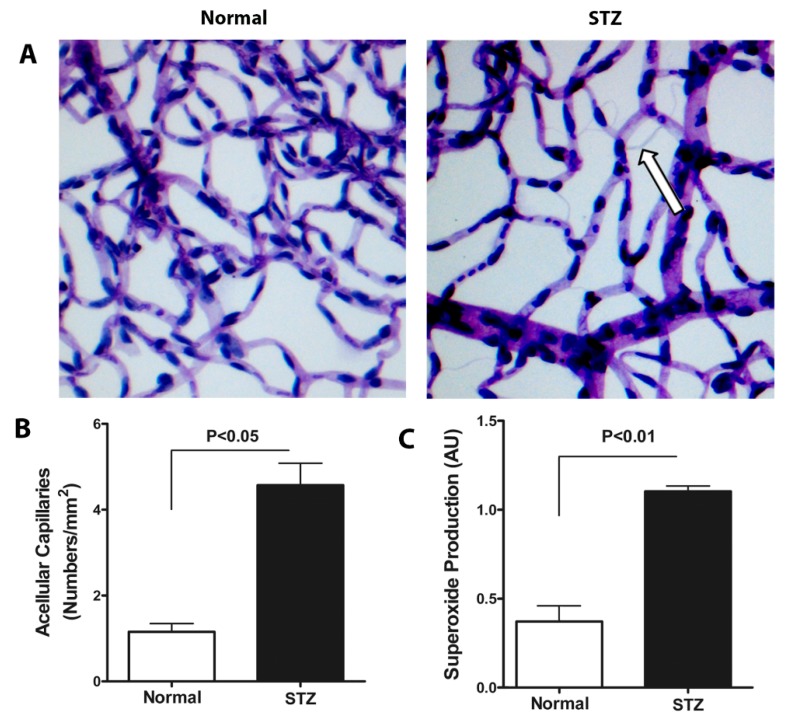
Evaluation of acellular capillary formation and superoxide anion generation in T1D mice. Representative images of trypsin-digested retinal vascular preparations from 4-month old control mice and 4-month old mice with 2-month duration of T1D (**A**) The arrow indicates an acellular capillary. Quantitative measurement of acellular capillaries was significantly increased in diabetic eyes (n = 6). (**B**) STZ-induced diabetic retina showed >1.5 fold increase in superoxide anion levels, *p* < 0.01 (**C**) The errors bars represent SEM.

Having established that autophagy in the retina possesses a diurnal rhythm, we next examined the rhythmicity of autophagy proteins in T1D. We chose mice from 2- and 9-months duration of diabetes to assess the expression of autophagic proteins. To confirm that our T1D mice exhibited the expected retinopathy features [36,37], we performed trypsin-digests of the retina from nondiabetic C57BL/6J (Figure 4A, left) and T1D (Figure 4A, right). T1D mice exhibited an increase in the number of acellular capillaries per unit area. Quantitative measurements of acellular capillaries suggested a dramatic 4-fold increase, in contrast to the age-matched nondiabetic mouse of retinas that showed a normal vascular pattern (Figure 4B). These eyes showed significantly higher levels of superoxide anions in the neural retina, typical of retinopathy in this model (Figure 4C).

**Figure 5:**
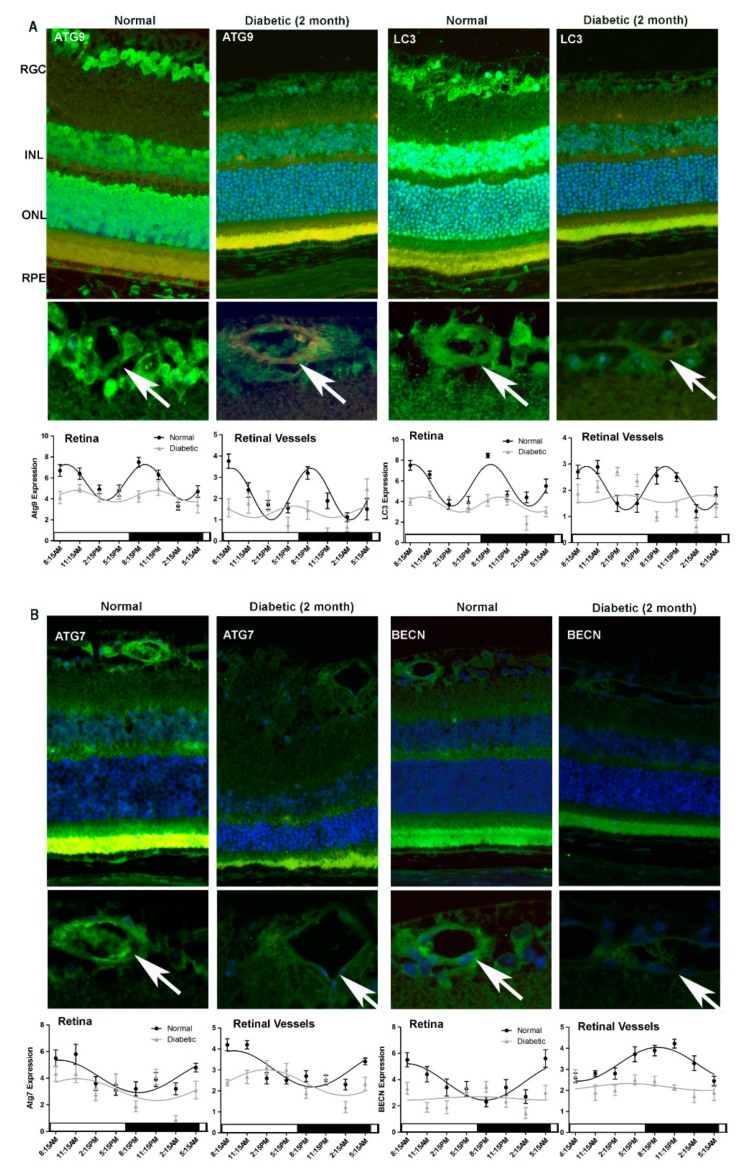
Impairment of diurnal rhythmicity of autophagy in T1D mice with two-months duration of diabetes. Retinas were collected from C57Bl/6J mice with two-months duration of T1D and age-matched control mice. Immunostaining and intensity analyses of retina and retinal vasculature demonstrated a dramatic loss of oscillatory amplitude of autophagic protein expression in the diabetic animals compared to normal mice (**A**,**B**). Autophagy protein expression is green, TRITC agglutinin for vessels red and DAPI nuclear staining blue. The arrows indicate autophagy protein localization to retinal vessels. RGC, retina ganglion cell; INL, inner nuclear layer; OPL, outer plexiform layer; ONL, outer nuclear layer; RPE, retinal pigment epithelium. Loss and phase-shifting of diurnal rhythmicity in diabetic retinopathy is demonstrated in single cosine plots, with ordinary least square fitted (*p* < 0.01, n = 10). All animals were maintained in a standard 12/12-h light/dark phase with lights ON at 6:00 AM and lights OFF at 6:00 PM. The error bars in the circadian plots represent the mean+SEM and diurnal oscillation had a *p*-value less than 0.05.

**Figure 6:**
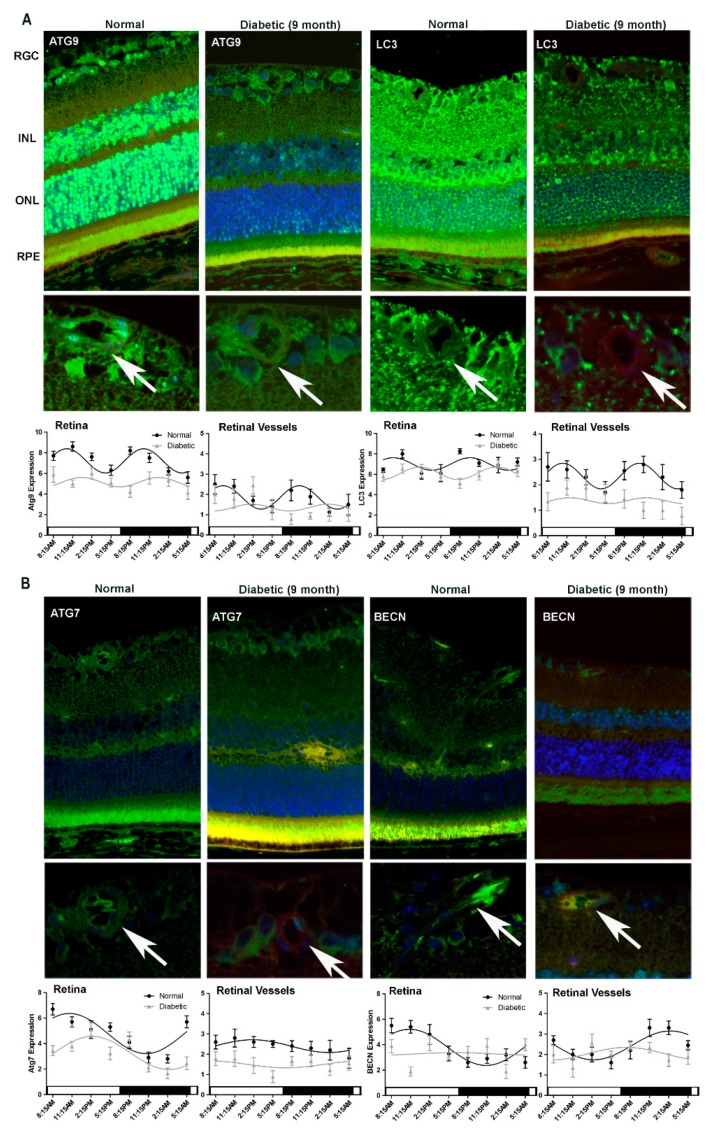
Impairment of diurnal rhythmicity of autophagy in T1D mice with nine-months duration of diabetes. Retinas were collected from C57Bl/6J mice with nine-months duration of T1D and age-matched control mice. Immunostaining and intensity analyses of retina and retinal vasculature demonstrated a dramatic loss of oscillatory amplitude of autophagic protein expression in the diabetic animals compared to normal mice (**A**.**B**). Autophagy protein expression is green, TRITC agglutinin for vessels red and DAPI nuclear staining blue. The arrows indicate autophagy protein localization to retinal vessels. RGC, retina ganglion cell; INL, inner nuclear layer; OPL, outer plexiform layer; ONL, outer nuclear layer; RPE, retinal pigment epithelium. Loss and phase-shifting of diurnal rhythmicity in diabetic retinopathy is demonstrated in single cosine plot with ordinary least square fitted (*p* < 0.01, n = 10). All animals were maintained in a standard 12/12-h light/dark phase with lights ON at 6:00 AM and lights OFF at 6:00 PM. The error bars in the circadian plots represent the mean+SEM and diurnal oscillation had a *p*-value less than 0.05.

While immunohistochemistry results from 4-month old normal control mice corresponded with the respective results shown in Figure 1 and Figure 2, we observed a dramatic attenuation of diurnal rhythmicity (amplitudes), as well as overall levels of the autophagic proteins in the T1D mice retina (Figure 5). This loss of amplitude was noted for ATG9, LC3 and Beclin1 expression in the diabetic retina, in either duration of diabetes group when compared to the respective age-matched controls (Figure 5 and Figure 6). ATG7 exhibited a shift in phase in both the retina and the vasculature of mice with 2-month duration of diabetes while in mice with 9-months duration of diabetes the phase-shift was prominent in the retina and there was no significant variation in expression between time-points in the retinal vasculature (Figure 5B and Figure 6B).

### 3.4. Autophagy Proteins Were Suppressed Severely in T2D Rats

**Figure 7:**
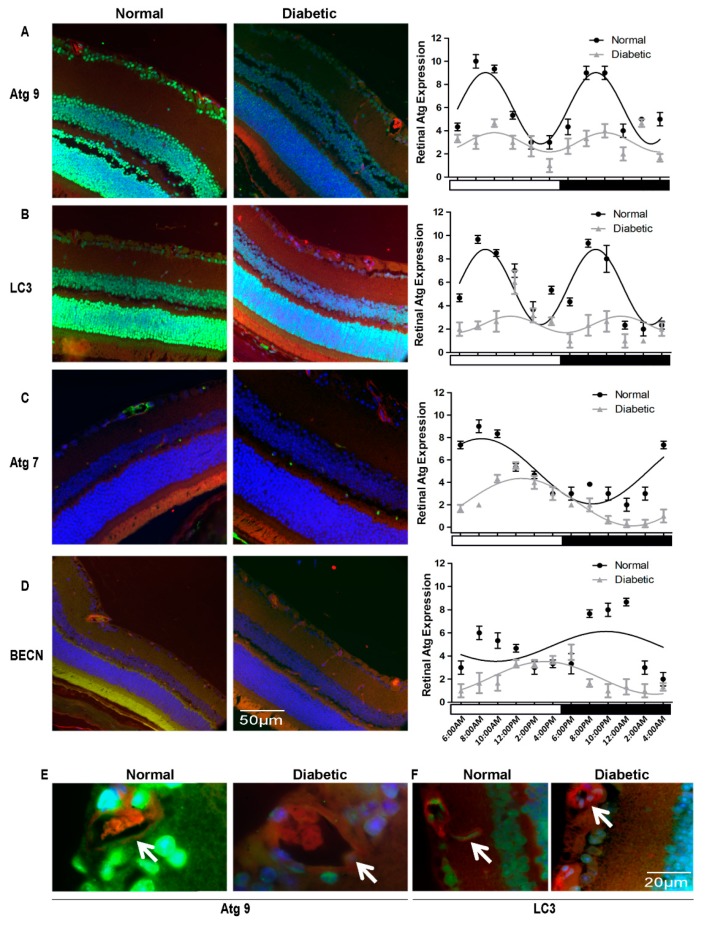
Impairment of diurnal rhythmicity of autophagy in T2D rats. Eyes were collected from 6.5-month old BBZDR/wor type 2 diabetic rats with 4-month duration of diabetes and the normal age-matched rats selected as controls. Immunostaining demonstrated a dramatic decrease in amplitude of diurnal rhythmicity of autophagic protein expression in the retina of the diabetic animals compared to normal rats. Representative micrographs of immunostained sections at 8:15 am are shown for (**A**) ATG9, (**B**) LC3 (**C**) ATG7 (**D**) Beclin1 (BECN). Autophagy protein expression is green, TRITC agglutinin for vessels red and DAPI nuclear staining blue. The arrows indicate autophagy protein localization to retinal vessels. RGC, retina ganglion cell; INL, inner nuclear layer; OPL, outer plexiform layer; ONL, outer nuclear layer; RPE, retinal pigment epithelium. ATG7 and Beclin1 displayed phase-shifting in the diurnal oscillation (**C**) and (**D**) respectively. Loss and phase-shifting of diurnal rhythmicity in diabetic retinopathy is demonstrated in single cosine plot with ordinary least square fitted (*p* < 0.01, n = 10). High magnification images confirmed the loss of autophagy proteins ATG9 and LC3 in the retinal vasculature (**E**). All animals were maintained in a standard 12/12-h light/dark phase with lights ON at 6:00 AM and lights OFF at 6:00 PM. The error bars in the circadian plots represent the mean + SEM and diurnal oscillation had a *p*-value less than 0.05.

We next examined autophagy in the retina of T2D rats following 4-months duration of diabetes (Figure 7). The normal age-matched rats selected as controls demonstrated a similar staining pattern of immunohistochemistry across the retina as were observed in normal mice (Figure 7A–D). Atg9 (Figure 7A) and LC3 (Figure 7B) were mainly present in the ganglion cell layer, inner and outer nuclear layers in the normal rat retina, similar to what was observed in normal mice (Figure 1) but their expression was dramatically decreased in the retinas of the diabetic animals by 49% to 58%, respectively. For better demonstration of changes in autophagic protein levels in the vasculature of diabetic retinas, we showed higher magnification images. Both Atg9 and LC3 clearly distributed within the retinal endothelia of normal rats but were completely absent from the endothelia of the diabetic retinas (Figure 7E,F). Atg7 was strongly expressed in normal retinal vessels near the ganglion cells layer, meanwhile the small vessels in inner and outer plexus layers were also stained (Figure 7C). Semi-quantitative analysis revealed that the level of Atg7 was deceased by 52%, *p* < 0.05 in diabetic retinas. Diabetic retina also displayed pathologic changes in retina vessels, which appeared to be abnormally protruded toward the intravitreous cavity. Beclin1 staining was detected across the retina, including retinal vessels in the plexus and photoreceptors, however, Beclin1 staining was diminished in diabetic retinas by 53%, *p* < 0.01 (Figure 7D). Quantitative analyses of autophagic proteins in the retina of control and diabetic mice demonstrated, not only suppressed levels, but also revealed impairment in diurnal rhythmicity in T2D rats. Expression of Atg9 and LC3 were severely suppressed with insignificant biphasic oscillatory pattern and ATG7 and Beclin1 were phase-shifted by approximately for 4–6 h. We concluded that disruption in autophagy is a characteristic phenomenon of both T1D and T2D.

## 4. Discussion

Autophagy is a critical and indispensable housekeeping process in the cells of the retina. Numerous reports support the functional relevance of autophagy in specific cells of the retina and how dysregulated autophagy contributes to retinal malfunction and degeneration [38,39,40,41,42,43]. While acute short-term noxious stimuli (e.g., nutrient deprivation; hypoxia and endoplasmic reticulum (ER) stress; oxidative stress arising from light, lipofuscin, POS phagocytosis by RPE or mitochondrial ROS generated in retinal cells (which have a generally high metabolic rate)) can stimulate autophagy as a generally cytoprotective mechanism, it must be acknowledged that diurnal basal autophagic modulation is a critical factor in abrogating the unavoidable regular cellular damage that occurs during the basic functioning of retinal cells. An efficient regulatory molecular circuit is required in the retina, that would modulate both the intensity and duration of basal autophagic activity in specific cell types and thus meet their regular housekeeping demands [44]. Previous studies have suggested the existence of autophagic rhythm in rodent heart muscles and liver, as well as kidney proximal tubules [45,46,47]. In this study, we establish the existence of diurnal regulation of autophagic activity, in both the neural and vascular cells of the retina. We show evidence that certain autophagic markers like Beclin1, ATG9 and LC3 are highly expressed across the retina, while ATG7 shows preferential staining patterns and is enriched only in certain layers (Figure 1 and Figure 2). The first report on diurnal rhythmicity in autophagy in the retina recorded increased autophagosome formation following outer disk shedding in rat photoreceptors [48]. Our results regarding the biphasic oscillation of diurnal rhythmicity of LC3 expression agree with the findings in a report by Yao et al. 2014, who also observed two peaks of elevated autophagic activity [29]. Our results in Figure 3 make a strong argument in favor of the influence of external stimuli such as light for the entrainment and maintenance of healthy amplitudes of oscillation of autophagic protein expression in the retina. However, we observed that even in the absence of light entrainment, the retina continued to display rhythmicity in ATG7, ATG9, LC3 and BECN, but were lower in amplitude and out of phase, suggesting that there may be an intrinsic diurnal rhythm of autophagy in the retina not dependent on light entrainment.

Our study encompasses autophagy in aging and diabetic conditions. We successfully demonstrate that perturbed autophagy is a characteristic feature of aging. It has been suggested that macroautophagy suffers a decline with aging and is replaced partly by other forms of autophagy [28]. It remains to be determined if other forms of autophagy i.e., the chaperone mediated autophagy and microautophagy are also under regulation by the circadian system and, if so, whether their rhythmicity is affected in aging and disease. In addition to the above, our study reveals that the level of disruption in T1D and T2D is significant, not only in younger mice, but also in older animals (Figure 5 and Figure 6). Compared to age-matched control mouse retinas, the dramatic deviations in the oscillatory patterns or the overall levels of one or more Atgs in both two- and nine-month old diabetic mouse retina could be an indication of faulty housekeeping and pathological outcomes, such as the accumulation of damaged mitochondria and ROS production [49,50,51,52].

A limitation of this study is that it relies on immunohistochemistry to determine changes in the expression of autophagy proteins throughout the neural retina. We did not attempt to confirm our findings by assessing gene expression or protein levels using Western blot. Neither did we assess autophagic flux by determining the LC3II:LC3I ratio. The reasons for this were a combination of the large number of animals needed; regional/cell-specific changes would be masked in whole retinal preps and the non-canonical role of LC3 in retinal and immune cell phagocytosis [25] could complicate the analysis of the autophagic flux. Furthermore, based on our data, there is a need to determine the mechanistic link between diurnal changes and autophagy in the neural retina. There is considerable evidence that diurnal/circadian rhythm is associated with the induction of autophagy [44,53], a key regulator of autophagy; the mechanistic target of rapamycin (mTOR) is regulated by the circadian clock [54] and the circadian regulation of metabolism is mediated through reciprocal signaling between the clock and metabolic regulatory networks such as autophagy [44]. Recently, Ryzhikov and colleagues reported that diurnal rhythms spatially and temporarily organize autophagy [55]. They reported that basal autophagy rhythms could be resolved into two antiphase clusters that were distinguished by the subcellular location of targeted proteins. Daytime autophagy was directed towards cytosolic proteins and proteosomal degradation, while nighttime autophagy was directed towards ER and mitochondrial removal. There is now a need to better understand the mechanistic control of these processes.

As mentioned above, autophagy in diabetes and related diseases has become an area of intense research, with focus on how this pathway may be targeted in synergy, with other therapeutic approaches to encourage a better clinical outcome. Our study, along with other recent reports, adds a novel extension to the mammalian diurnal rhythmicity in its relevance in regulation of biological processes. It provides a novel link between dysregulated autophagy and disrupted diurnal rhythm in the aging and diabetic retina and suggests that our analyses of myriad biological processes in the retina should be reconsidered from a diurnal perspective, in order to better comprehend age-related vision loss and disease pathology.
